# The Prognostic Value of Locoregional Interventions for BRAF V600E Metastatic Colorectal Cancer: A Retrospective Cohort Analysis

**DOI:** 10.3390/biom11091268

**Published:** 2021-08-25

**Authors:** Liu-Fang Ye, Xiao-Meng Ji, Chao Ren, Zhi-Qiang Wang, Chun-Ping Lin, Dong-Liang Chen, Yan-Qing Cai, Ying Jin, Miao-Zhen Qiu, Zi-Ming Du, Shao-Yan Xi, Dong-Sheng Zhang, Feng Wang, Feng-Hua Wang, Rui-Hua Xu, Yu-Hong Li, De-Shen Wang

**Affiliations:** 1 Collaborative Innovation Center for Cancer Medicine, Sun Yat-sen University Cancer Center, State Key Laboratory of Oncology in South China, Sun Yat-sen University, Guangzhou 510060, China; yelf@sysucc.org.cn (L.-F.Y.); renchao@sysucc.org.cn (C.R.); wangzhq@sysucc.org.cn (Z.-Q.W.); chendl@sysucc.org.cn (D.-L.C.); jinying1@sysucc.org.cn (Y.J.); qiumzh@sysucc.org.cn (M.-Z.Q.); zhangdsh@sysucc.org.cn (D.-S.Z.); wangfeng@sysucc.org.cn (F.W.); wangfh@sysucc.org.cn (F.-H.W.); xurh@sysucc.org.cn (R.-H.X.); 2 Research Unit of Precision Diagnosis and Treatment for Gastrointestinal Cancer, Chinese Academy of Medical Sciences, Guangzhou 510060, China; 3 Department of Medical Oncology, Sun Yat-sen University Cancer Center, Guangzhou 510060, China; 4 Department of Molecular Diagnostics, Sun Yat-sen University Cancer Center, Guangzhou 510060, China; jixm@sysucc.org.cn (X.-M.J.); duzm1@sysucc.org.cn (Z.-M.D.); 5 Department of Oncology, Jieyang Affiliated Hospital, Sun Yat-sen University, Jieyang 522000, China; jylcpd@163.com (C.-P.L.); cai99yq@126.com (Y.-Q.C.); 6 Department of Pathology, Sun Yat-sen University Cancer Center, Guangzhou 510060, China; xishy@sysucc.org.cn

**Keywords:** BRAF V600E, metastatic colorectal cancer, prognosis, heterogeneity, locoregional interventions

## Abstract

The prognostic heterogeneity in patients with BRAF V600E metastatic colorectal cancer (mCRC) remains poorly defined. Real-world data of 93 BRAF V600E mCRC patients from Sun Yat-sen University Cancer Center were evaluated using the prognostic factors affecting overall survival (OS). Treatment of metastases served as an independent prognosticator, where curative locoregional interventions (LRIs) were associated with superior clinical outcomes (adjusted hazard ratio (HR): 0.46, 95% confidence interval (CI): 0.22–0.98; *p* = 0.044). The LRIs group showed an improved median OS of 49.4 months versus 18.3 months for the palliative treatments (PTs) group. The median OS of patients with colorectal liver metastasis (CRLM) was significantly prolonged after undergoing LRIs (42.4 vs. 23.7 months; HR: 0.11, 95% CI: 0.01–1.22; *p* = 0.030), and patients in the LRIs plus liver-limited or lung-limited metastasis (LLM) group benefited more than those in the LRIs plus non-LLM group when compared to the PTs group (LLM from LRIs vs. PTs, HR: 0.16, 95% CI: 0.04–0.68; *p* = 0.006. Non-LLM from LRIs vs. PTs, HR: 0.47, 95% CI: 0.21–1.05; *p* = 0.074). In conclusion, we confirmed the positive prognostic value of LRIs in BRAF V600E mCRC, particularly in patients with CRLM or LLM.

## 1. Introduction

Colorectal cancer (CRC) is the third most common malignancy, and its incidence has shown an increasing trend in developing countries [1,2]. In China, about 376.3 thousand new CRC cases were reported in 2015, with a prevalence of 57.3% and 42.7% in males and females, respectively [3]. CRC patients often present with metastatic disease at the time of diagnosis, decreasing the five-year overall survival (OS) for these patients to only 12%, and those harboring oncogene mutations have even worse clinical outcomes [4,5,6].

BRAF mutations, one of the most well-studied oncogene mutations, are present in approximately 7–11% of metastatic CRC (mCRC) cases [7,8,9,10]. The BRAF V600E mutation, which accounts for nearly 80% of BRAF mutations, often exhibit aggressive features and is associated with elderly persons, female gender, right-sided primary, advanced stage, high grade and peritoneal dissemination. Interestingly, studies show that BRAF V600E mutation cases have a worse clinical outcome than those with BRAF mutations other than V600E or wild-type BRAF [11,12]. Moreover, in the metastatic setting, BRAF V600E mutation is a negative indicator of prognosis and might be a driver of the early resistance to current treatment strategies [13,14]. In contrast to BRAF wild-type patients, BRAF-mutant patients are more likely to encounter rapidly deteriorating health after first-line therapy progressed, and nearly 40% of them fail to receive subsequent treatments, hence the need to strengthen initial treatment decisions in these patients [6]. Indeed, multimodal therapies were proposed and integrated into their treatment, particularly those with limited metastatic dissemination [15,16]. Although the survival outcomes are generally poor compared with wild-type BRAF mCRC, some patients can survive up to 5 years after undergoing metastasectomy [17]. A multi-institutional study from 1497 patients with colorectal liver metastases (CRLMs) showed that 17% of BRAF mutant cases benefited from long-term OS following surgical intervention [18]. Consistently, patients with BRAF V600E mCRC also displayed significantly improved OS after metastasectomy compared to those who did not, and another real-world study also revealed a similar survival advantage of incorporating metastasectomy in multimodality therapy [19,20].

The prognostic heterogeneity of patients with BRAF V600E mCRC might be explained by differences in clinical characteristics and treatment features. Therefore, it is essential to apply these factors to the prognostic analyses for BRAF V600E mCRC. Previous publications reported that the clinical characteristics of patients could be related to different survival outcomes for BRAF V600E mCRC. For instance, Loupakis et al. established a prognostic scoring system that classified patients into three different prognostic subgroups based on clinical, pathological, and laboratory factors to better stratify patients with the BRAF V600E mutation [21]. Although Loupakis et al. further explored the prognostic value in deciding the intensity of first-line systemic chemotherapy, patients’ treatment goals and the use of local therapy in the first-line setting were poorly described. This is vital since locoregional treatments with curative intent integrated into first-line therapy are themselves positive prognostic determinants associated with a decreased risk of death [22]. Despite recent advances, the heterogeneity in survival results remains poorly defined for patients with the BRAF V600E mutation, hence the need for an in-depth study. 

The study aims to provide real-world data from a retrospective China cohort and identify the prognostic factors for OS in patients with BRAF V600E mCRC. We hypothesized that the heterogeneity of clinical outcomes in the BRAF V600E mCRC population might be due to diverse clinical characteristics and treatment features such as the choice of initial therapy, in which therapeutic intent and use of local therapy were added to the management of their treatment strategies, and if so, the administration of radical local therapy in first-line setting may correlate with better prognosis in this population. This study reveals that the addition of a first-line local curative therapy was associated with a better prognosis profile in BRAF V600E mCRC patients. More importantly, this correlation was even more pronounced in patients with CRLM or liver-limited or lung-limited metastasis (LLM) who had locoregional interventions (LRIs).

## 2. Materials and Methods

### 2.1. Patient Selection and Data Collection

**Figure 1:**
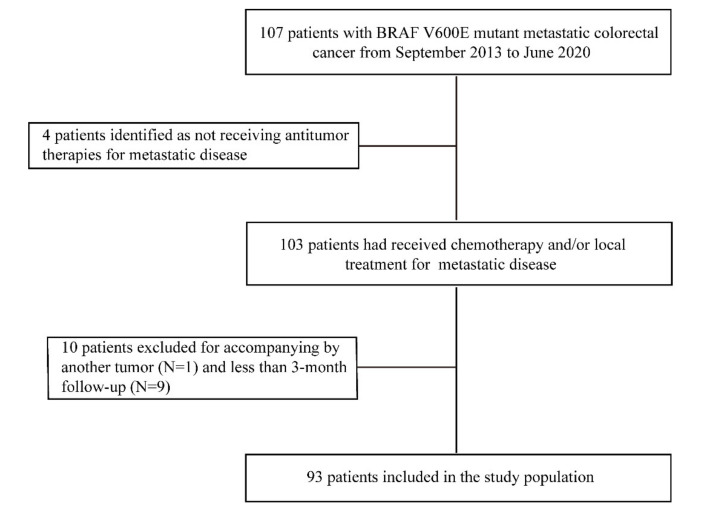
Flow chart of patient selection of this study.

We retrospectively identified 107 consecutive patients documented as having BRAF V600E mCRC from September 2013 to June 2020 at Sun Yat-sen University Cancer Center. The BRAF mutation status was tested using sanger sequencing or next-generation sequencing (NGS). The flow chart of patient selection is shown in Figure 1. Of the 107 patients enrolled, we included those who had received antitumor therapies for metastatic disease, e.g., chemotherapy or local treatment, either alone or in combination (*N* = 103). Patients were excluded if they had another tumor at the diagnosis of metastases (*N* = 1) or less than a 3-month follow-up (*N* = 9). In the end, a total of 93 mCRC patients with the BRAF V600E mutation were included in the study population. We collected data from the medical records of 93 patients, including demographic data, clinical and pathological characteristics, treatment features, and survival outcomes. Mismatch repair (MMR) or microsatellite instability (MSI) status and genetic variations of RAS genes were also collected. This retrospective study was approved by the Ethical Committee of Sun Yat-sen University Cancer Center (protocol code GZR2020-222, approved on 11 March 2020).

### 2.2. Treatment Features and Definitions

All patients were assessed and classified into LRIs group and palliative treatments (PTs) group based on the intent of their treatment. The LRIs group comprised of patients receiving treatment with curative intent whereby all identifiable lesions for both the primary tumor and metastases were eliminated through surgical R0/R1 resection (resection of all visible lesions with or without the presence of microscopic residual tumor) and/or appropriate non-surgical treatments such as radiofrequency/microwave ablation (RFA/MWA), cryoablation, stereotactic body radiotherapy (SBRT), or chemoembolization, either initially or possibly after effective systemic therapy [15]. For patients with colorectal peritoneal metastasis (CRPM), cytoreduction surgery (CRS) with or without hyperthermic intraperitoneal chemotherapy (HIPEC) was been considered for selected patients with limited peritoneal tumors [16,23]. Those who have eradicated all macroscopical tumor masses receiving CRS with or without HIPEC were also included in the LRIs group. PTs group were defined as those who received systemic therapy and/or local palliative treatment.

Except for demographic and clinicopathologic features, detailed information on metastases was obtained for the comparative analysis between different treatment groups, such as metastatic disease type (synchronous (<6 months) vs. metachronous), metastatic site (the first documented anatomic organ(s) with metastases), number of organs involved, and oligometastatic state (oligometastatic disease (OMD) vs. non-OMD; the former referring to disease in which a maximum total of five lesions were present in up to three organs other than the central nervous system, bone, ascites and peritoneum). Patients with no more than three metastatic lesions per organ with a maximum size of 3 cm were categorized as having OMD/low tumor burden (TB) [22]. Variables in the LRIs group, including the type of LRIs, therapeutic features, and disease recurrence, were also recorded.

OS was calculated from the date of diagnosis of metastases to the date of death or last follow-up. Disease-free survival (DFS) was calculated from the date of LRIs administration to the date of the first documented recurrence or the date of the last follow-up if no disease recurrence was found. Survival after recurrence (SAR) was calculated for patients who experienced disease recurrence after LRIs and was defined as the interval from the date of recurrence to the date of death or last follow-up.

### 2.3. Statistical Analysis

Differences in categorical variables between different groups were analyzed with the Chi-square test or Fisher’s exact test. Probabilities of time-to-event variables (OS, DFS and SAR) were calculated using the Kaplan–Meier method, and the log-rank test compared differences in survival curves. Univariate Cox proportional hazards analysis was used to estimate hazard ratios (HRs) and 95% confidence intervals (CIs) of potential prognostic factors, and independent prognostic factors were assessed using multivariate Cox analysis. A two-sided *p* value of 5% was set as the cutoff for statistical significance. Analyses were carried out using SPSS software (version 26.0, IBM) and R software (version 4.0.2, R Foundation for Statistical Computing).

## 3. Results

### 3.1. Study Population

A total of 93 patients with the BRAF V600E mutation were identified in the current study (Figure 1). As shown in Table 1, the median age of patients was 52.0 years, and 66.7% of them were male. About three-quarters of patients (73.1%) underwent primary tumor resection, with 22 patients (23.7%) receiving adjuvant therapy. Common malignant biological characteristics in BRAF V600E mCRC patients were also observed, including advanced T4 stage (50.6%), positive lymph nodes (82.8%), and synchronous metastases (76.3%). About half of patients (49.5%) had one metastatic organ involvement, and OMD was defined in 11 patients (12.0%). Twenty patients (21.5%) had a liver-limited or lung-limited disease. Thirty-two patients (34.4%) were treated with triplet chemotherapy as first-line therapy, and forty-eight patients (51.6%) received bevacizumab in the first-line setting. LRIs with curative intent were administrated to a total of 32 patients (34.4%), while the remainder received PTs. Three patients (3.2%) harbored concomitant RAS mutation, and one patient (1.1%) had deficient MMR (dMMR).

### 3.2. OS Analysis

**Figure 2:**
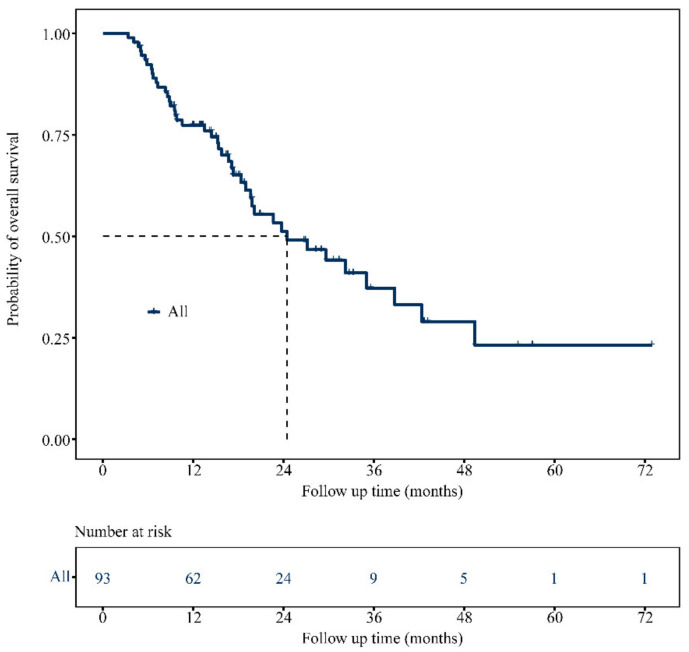
Kaplan–Meier analysis of OS in all mCRC patients with the BRAF V600E mutation.

With a median follow-up of 26.7 months (95% CI: 17.1–36.3), the 93 patients with BRAF V600E mCRC had a median OS of 24.5 months (95% CI: 13.9–35.0; Figure 2), with 1- and 3-year OS rates of 77.4% and 37.3%, respectively. 

In the multivariate analysis, when controlling for other covariates that might have potentially affected OS, we found that treatment of metastases was significantly and independently associated with prognosis (adjusted HR: 0.46, 95% CI: 0.22–0.98; *p* = 0.044; Table 2). 

**Figure 3:**
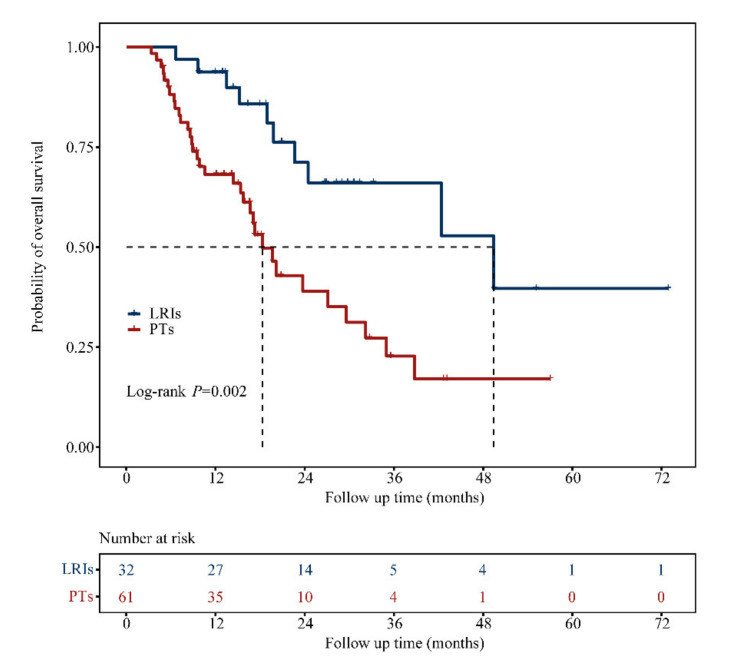
Kaplan–Meier analysis of OS in mCRC patients with the BRAF V600E mutation who received different treatments. Abbreviations: LRIs, locoregional interventions; PTs, palliative treatments.

**Figure 4:**
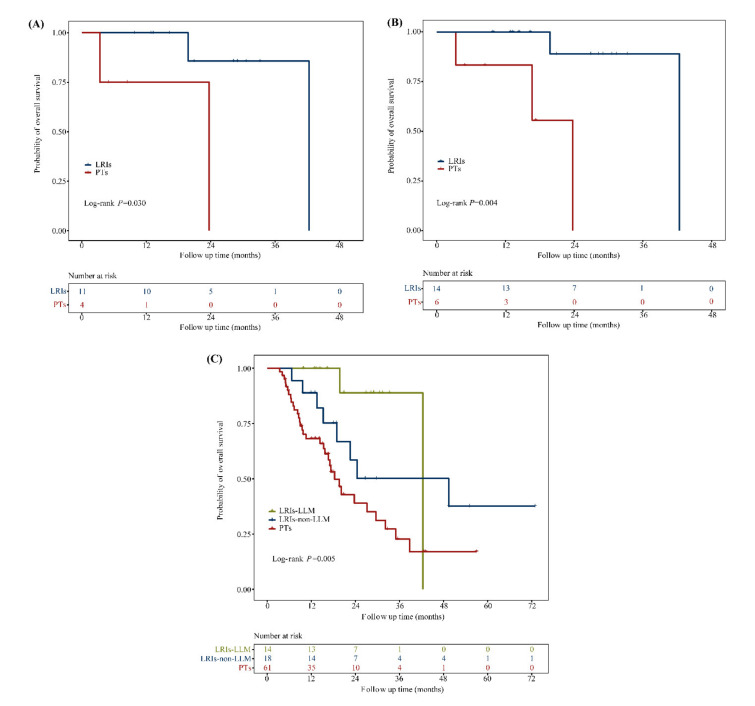
Kaplan–Meier analysis of OS in mCRC patients with the BRAF V600E mutation. (**A**) CRLM cohort, LRIs for patients with CRLM vs PTs for patients with CRLM, median OS: 42.4 vs 23.7 months, HR: 0.11, 95% CI: 0.01–1.22, *p* = 0.030. (**B**) LLM cohort, HR: 0.07, 95% CI: 0.01–0.69, *p* = 0.004. (**C**) Three subgroups of LLM from LRIs, non-LLM from LRIs and PTs, respectively. LRIs for patients with LLM vs. PTs, HR: 0.16, 95% CI: 0.04–0.68, *p* = 0.006. LRIs for patients with non-LLM vs. PTs, HR: 0.47, 95% CI: 0.21–1.05, *p* = 0.074. Abbreviations: LRIs, locoregional interventions; PTs, palliative treatments; LLM, liver-limited or lung-limited metastasis; LRIs-LLM, patients with LLM received LRIs; LRIs-non-LLM, patients who were not LLM received LRIs.

Patients who underwent LRIs showed significantly longer OS than those treated with PTs (49.4 vs. 18.3 months; HR: 0.34, 95% CI: 0.17–0.70; *p* = 0.002; Figure 3). The OS rates at 1 and 3 years were 93.8% and 66.1%, respectively, in the LRIs group versus 68.2% and 22.7% in the PTs group. For patients with CRLM, the survival benefit provided by LRIs was more apparent than that provided by PTs (42.4 vs. 23.7 months; HR: 0.11, 95% CI: 0.01–1.22; *p* = 0.030; Figure 4A). LRIs for patients with LLM exerted a positive effect on OS (HR: 0.07, 95% CI: 0.01–0.69; *p* = 0.004; Figure 4B) and appeared to be more beneficial than LRIs for patients with non-LLM when similarly compared to patients treated with PTs (LRIs for patients with LLM vs. PTs, HR: 0.16, 95% CI: 0.04–0.68; *p* = 0.006. LRIs for patients with non-LLM vs. PTs, HR: 0.47, 95% CI: 0.21–1.05; *p* = 0.074. Figure 4C).

### 3.3. Clinical Characteristics between Treatment Groups

Clinicopathological and metastatic features of treatment groups are presented in Table 3. Primary tumor features and metastasis data showed significant differences between treatment groups. Primary tumor resection (*p* < 0.001) and negative lymph nodes (*p* = 0.043) were more common in the LRIs group. Patients in the LRIs group experienced more isolated metastasis than those in the PTs group, whereas a large proportion of patients in the PTs group had two or more metastatic sites (*p* < 0.001). In terms of metastatic location(s), compared with those in the PTs group, patients in the LRIs group had more frequent liver-limited or lung-limited involvement (*p* < 0.001), including ten patients with OMD while only one patient in the PTs group had OMD (*p* < 0.001). In addition, peritoneal-only metastasis was also more commonly observed in the LRIs group than in the PTs group (*p* = 0.005). In patients with two or more metastatic sites, fewer patients with multiple non-peritoneal metastases were observed in the LRIs group than in the PTs group (*p* < 0.001). Although the proportion of non-isolated peritoneal metastases difference was not pronounced between treatment groups (*p* = 0.092), there were more patients with peritoneal metastasis and metastasis in two or more other sites in the PTs group than in the LRIs group (*p* = 0.021). 

### 3.4. Focus on the LRIs Group

As presented in Supplementary Table S1, twenty-seven patients (84.4%) received surgical resection, and five patients (15.6%) received non-surgical therapies; both strategies were applied with curative intent. Approximately forty-five percent of patients had received chemotherapy before receiving LRIs, and eighty-four percent of patients had received adjuvant chemotherapy after LRIs, including one-, two- or three-drug regimens. The median DFS was 12.4 months (95% CI: 4.7–20.1), and the 1-year DFS rate was 51.5%, as shown in Figure S1. Twenty-four patients (77.4%) experienced recurrent disease, with multisite as the initial recurrence occurring in more than half (54.2%) (Supplementary Table S1) and recurrence only involving liver or lung observed in seven patients (29.2%). After recurrence, the median OS was only 17.6 months (95% CI: 6.9–28.2; Figure S1). Of patients with LLM (Supplementary Table S2), ten of fourteen patients (71.4%) had OMD, including six (42.9%) patients with OMD/low TB. Ten of fourteen patients (71.4%) received systemic chemotherapy before LRIs, six of whom (60%) were treated with fluorouracil, leucovorin, irinotecan, and oxaliplatin (FOLFOXIRI) plus bevacizumab and the others (40%) were in the doublet chemotherapy regimen. Five of these fourteen patients had no evidence of disease recurrence at the end of follow-up. Of those, one patient remained relapse-free for more than two years, and another showed nearly two years of relapse-free survival after effective chemotherapy.

## 4. Discussion

Herein, we demonstrated that curative intent LRIs positively impacted survival outcomes and was an independent factor affecting the prognosis of BRAF V600E mCRC patients. The rationale of LRIs possibly resulted from a higher proportion of patients with limited metastases in this subgroup. LRIs provided a survival advantage among patients with CRLM compared with chemotherapy and/or local palliative treatment. Specifically, those patients with LLM seemed to benefit the most from LRIs, having a markedly decreased risk of poor outcomes. Nevertheless, interpretation of these two findings should be made with caution due to the small sample size. Importantly, our study suggests that it is appropriate to integrate curative intent LRIs into the treatment strategy for BRAF V600E mutation mCRC patients to improve their survival despite the commonly attributed high recurrence rates and worse post-recurrence survival after LRIs.

The median OS (24.5 months) of patients with the BRAF V600E mutation in our cohort was longer than those previously reported for mCRC patients with this mutation (range: 10.1–15.5 months) [24], reflecting the high proportion of patients who received LRIs in our study. We identified 32 patients with the BRAF V600E mutation treated with LRIs, who accounted for 34.4% of the study population. Moreover, the LRI was a significant prognostic factor in our multivariate analysis, in which LRI was shown to confer a lower risk of death. Our results indicate that the LRIs group and PTs have different clinical characteristics. Compared with the PTs group, patients in the LRIs group were likely to have primary lymph node negativity and single-organ involvement; they displayed an increased rate of LLM, indicating that patients with limited metastasis might be eligible for a more aggressive treatment strategy. Therefore, it is legitimate for this subset of patients to receive LRIs due to these favorable factors. LRIs resulted in a median OS of 49.4 months compared with 18.3 months for PTs in our study. Our results are consistent with the report by Fouchardière et al., which suggested a similar median OS of 47.4 months for BRAF-mutant patients undergoing metastasectomy compared with 19.5 months for those who did not [25]. Additionally, Morris et al. reported that favorable survival in BRAF V600E mCRC was partly attributed to definitive locoregional therapy of metastatic disease and/or chemotherapy [26]. Taken together, our findings identify the important role of LRIs among patients exhibiting the BRAF V600E mutation, and these findings can help improve survival outcomes.

In the present study, approximately 12% of the patients in our cohort were BRAF V600E CRLM patients who underwent surgical resection and/or received other treatment modalities of liver metastasis, and we proved that elimination of all visible lesions with curative intent yielded survival improvement in patients with this mutation; 81.8% of patients received chemotherapy before LRIs and were assessed as having a radiological response or stable disease. According to two recent systemic reviews focused on patients with CRLMs, mutant BRAF appeared to negatively impact OS after resection compared to wild-type BRAF; thus, patients with BRAF V600E mutation were considered a poor fit for surgical therapy [27,28]. However, Cloyd et al. demonstrated a better median OS for CRLM patients with BRAF V600E mutation who were treated by radical resection than for those treated with chemotherapy alone, and Gagnière et al. identified a long-term clinical benefit from surgery, especially for those patients with favorable factors, thus suggesting that surgical resection is justified for some patients [18,29]. Our findings reinforce the clinical implications of multidisciplinary treatments for CRLM patients with the BRAF V600E mutation, further clarifying these contradictory results.

As implied by European Society for Medical Oncology (ESMO) consensus guidelines, LRIs were often considered more likely to induce long-term survival in patients with LLM [15]. This was confirmed in our study population, in which such patients showed prolonged survival and the lowest HR for OS than those treated with PTs. Approximately 75% of patients in the LLM plus LRIs group were classified as having OMD, defined as a metastatic disease that has spread to a limited number of regions with the absence of metastases in the central nervous system, bone, ascites and the peritoneum [22]. The favorable survival results following LRIs in this subgroup of patients might reflect the less aggressive molecular features of OMD, which likely represents a new subtype of the disease, carrying more favorable molecular characteristics that correlate with improved survival [30]. Moreover, local treatment of OMD provided the greatest likelihood of long-term disease control or was even curative for some mCRC patients [22]. However, only one patient in the PTs group had an OMD phenotype, limiting further exploration and comparison between the LRIs and PTs groups in OMD patients.

Intriguingly, we found a proportion of patients with peritoneum-confined involvement in the LRIs group, half of which had an OS of more than 2 years. Recent studies have reported the improved survival of CRC patients with peritoneal carcinomatosis that received CRS with or without HIPEC. Furthermore, a long-term survival duration of up to 63 months was achieved following surgery in patients with isolated and resectable peritoneal tumors [31,32]. Nonetheless, in our cohort, these data on the peritoneal carcinoma index were not available to determine those patients with the BRAF V600E mutation who are most likely to benefit from resection of peritoneal carcinomatosis, as has previously been confirmed the prognostic value of OS [33].

Despite a high rate of disease recurrence, a subset of patients with the BRAF V600E mutation might benefit from long-term DFS, especially patients with LLM. Nearly three-fourths of patients with LLM received systemic chemotherapy before LRIs, including 60% in the tri-chemotherapy and 40% in the bi-chemotherapy, all of whom had a response or stable disease, although OS was not significantly different between the bi-chemotherapy and tri-chemotherapy in our study. However, the median DFS of 12.4 months in our cohort was longer than that reported by Johnson et al., with an incremental benefit of 2.7 months, which could be explained due to the increased use of combination treatment FOLFOXIRI plus bevacizumab before LRIs in our study [20]. Triplet chemotherapy plus bevacizumab was associated with an increased rate of conversion surgery, with an R0 resection rate approaching 30%; such treatment showed an improved recurrence-free interval in previous publications, thus implying the importance of such an intensive regimen in a perioperative setting [34,35]. Recent meta-analysis data has found that FOLFOXIRI plus bevacizumab provided an advantage in progression-free survival, objective response rate, and R0 resection rate and significantly improved survival of patients with mCRC compared with doublets plus bevacizumab. However, no increased benefit was observed among patients with the BRAF V600E mutation tumors [24]. Therefore, whether FOLFOXIRI plus bevacizumab should be the preferable upfront option and which subgroup of patients could benefit from the intensified approach need further investigation. Nevertheless, we found a poor median survival of 17.6 months after relapse, consistent with the results observed in patients with resected stage III or IV colorectal tumors [36,37]. The poor OS after relapse might have resulted from the fact that more than half of patients often develop the recurrent disease at multiple sites, which was confirmed to be an indicator of poor survival after recurrence [38]. Moreover, the rate of liver-limited or lung-limited recurrence reached almost 30%, suggesting that an aggressive adjuvant therapeutic strategy, for example, the intra-arterial chemotherapy, should therefore be developed to prevent post-LRIs recurrence, and repeated LRIs are likely to improve the survival of such patients after a novel effective systemic treatment.

We recognize that our current study has the following limitations. Firstly, the present analysis was retrospective and analyzed by a single-center cohort; in addition, we only focused on patients with the BRAF V600E mutation, which might have resulted in selection bias. Secondly, the number of patients with CRLM or LLM in our center was small, and thus, the results should be interpreted with caution. Thirdly, MMR or MSI status data were insufficient to analyze the mismatch repair system in patients with the BRAF V600E mutation; however, different survival outcomes were noted in advanced and mCRC with the BRAF V600E mutation [39,40]. Besides, in this cohort, we only identified one patient (1.1%) as dMMR, which is much lower than in western countries [41]. Therefore, further comparative studies between countries are needed to distinguish whether the clinical characteristics and molecular events in patients with such mutations are also different. Lastly, several molecular features, such as the consensus molecular subtypes (CMSs), were not factored into the prognosis analysis; those were recently confirmed as one of the major prognosticators in BRAF V600E mCRC [42]. Regardless of these limitations, our study offers a fresh perspective on the management of BRAF V600E mCRC patients. Further prospective cohorts are warranted to confirm our results to help oncologists stratify those eligible to receive LRIs and ultimately improve clinical outcomes.

## 5. Conclusions

In conclusion, we reveal that patients with BRAF V600E mCRC showed survival advantages after undergoing curative LRIs. Moreover, we also observed a subset of BRAF V600E mutation patients with liver-limited involvement that showed survival benefit after LRIs. Patients with LLM appeared to benefit more from LRIs than those with non-LLM when compared with PTs. In light of our findings, we believe that it is important to implement a more intensive treatment strategy in BRAF V600E mutation mCRC patients undergoing LRIs to prevent the recurrence since failure to do so will ultimately lead to progression and a worst overall survival.

## Figures and Tables

**Table 1:** Clinicopathological characteristics and therapeutic features of mCRC patients with the BRAF V600E mutation in our cohort.

Characteristics		No. of Patients (%)
		Overall (*N* = 93)
Age at metastases	Median, range	52.0 (14.0–79.0)
Gender	Male	62 (66.7)
	Female	31 (33.3)
Primary tumor site	Left	52 (55.9)
	Right	39 (41.9)
	Multisite	2 (2.2)
Differentiation	Well or moderate	66 (71.0)
	Poor	26 (27.9)
	Unknown	1 (1.1)
Primary tumor resected	Yes	68 (73.1)
	No	25 (26.9)
Primary T stage	T3	31 (33.3)
	T4	47 (50.6)
	Tx	15 (16.1)
Primary lymph node status	Positive	77 (82.8)
	Negative	16 (17.2)
Previous adjuvant chemotherapy	Yes	22 (23.7)
	No	71 (76.3)
Metastatic disease	Synchronous	71 (76.3)
	Metachronous	22 (23.7)
Number of organs involved	<2	46 (49.5)
	≥2	47 (50.5)
Oligometastatic state ^a^	OMD	11 (12.0)
	Non-OMD	81 (88.0)
Liver or lung metastasis only	Yes	20 (21.5)
	No	73 (78.5)
Peritoneal metastasis only	Yes	22 (23.7)
	No	71 (76.3)
Multiple non-peritoneal metastases ^b^	Yes	26 (28.0)
	No	67 (72.0)
First-line chemotherapy	Tri-chemo	32 (34.4)
	Bi-chemo	54 (58.1)
	Others	3 (3.2)
	Unknown	4 (4.3)
Bev in first-line chemotherapy	Yes	48 (51.6)
	No	41 (44.1)
	Unknown	4 (4.3)
Treatment of metastases	LRIs	32 (34.4)
	PTs	61 (65.6)
RAS status	Mutant	3 (3.2)
	Wide-type	85 (91.4)
	Unknown	5 (5.4)
MMR/MSI status	dMMR/MSI-H	1 (1.1)
	pMMR/MSS	86 (92.5)
	Unknown	6 (6.4)

^a^ One patient was not evaluable due to oligometastatic state. ^b^ Multiple non-peritoneal metastases: Combination of two or more metastatic organs, including liver, lung, distant lymph node or other non-peritoneal sites. Abbreviations: OMD, oligometastatic disease; Tri-chemo, tri-chemotherapy; Bi-chemo, bi-chemotherapy; Bev, bevacizumab; LRIs, locoregional interventions; PTs, palliative treatments; MMR, mismatch repair; MSI, microsatellite instability; dMMR, deficient MMR; MSI-H, high MSI; pMMR, proficient MMR; MSS, microsatellite stable.

**Table 2:** Univariate and multivariate Cox proportional hazards analysis of overall survival for mCRC patients with the BRAF V600E mutation.

Variables	*N*	Univariate Cox Analysis			Multivariate Cox Analysis		
		HR	95% CI	*p* Value	HR	95% CI	*p* Value
Age at metastases (<65 vs. ≥65)	76/17	1.19	0.55–2.59	0.666			
Gender (Male vs. female)	62/31	0.79	0.40–1.54	0.485			
Primary tumor site (Left vs. right)	52/39	0.87	0.46–1.65	0.667			
Differentiation (Well or moderate vs. poor)	66/26	1.14	0.58–2.23	0.705			
Primary tumor resected (Yes vs. no)	68/25	1.93	0.94–3.98	0.075			
Primary lymph node status (Negative vs. positive)	16/77	0.90	0.40–2.06	0.809			
Previous adjuvant chemotherapy (Yes vs. no)	22/71	1.41	0.67–2.94	0.366			
Metastatic disease (Metachronous vs. synchronous)	22/71	1.80	0.80–4.05	0.155			
Number of organs involved (<2 vs. ≥2)	46/47	2.05	1.11–3.79	0.023			
Oligometastatic state (Non-OMD vs. OMD)	81/11	0.13	0.02–0.97	0.046			
Liver or lung metastasis only (Yes vs. no)	20/73	2.36	0.92–6.02	0.073			
Peritoneal metastasis only (Yes vs. no)	22/71	1.79	0.85–3.76	0.126			
Multiple non-peritoneal metastases (Yes vs. no)	26/67	0.70	0.36–1.37	0.296			
First-line chemotherapy (Bi-chemo or others vs. tri-chemo)	57/32	0.97	0.48–1.97	0.931			
Bev in first-line treatment (Yes vs. no)	48/41	1.37	0.73–2.58	0.333			
Treatment of metastases (PTs vs. LRIs)	61/32	0.34	0.17–0.70	0.003	0.46	0.22–0.98	0.044

Abbreviations: OMD, oligometastatic disease; Bi-chemo, bi-chemotherapy; Tri-chemo, tri-chemotherapy; Bev, bevacizumab; PTs, palliative treatments; LRIs, locoregional interventions; HR, hazard ratio; CI, confidence interval.

**Table 3:** Differences of clinical characteristics between treatment groups in BRAF V600E mCRC patients.

Characteristics		No. of Patients (%)		
		PTs (*N* = 61)	LRIs (*N* = 32)	*p* Value
Age at metastases	<65	50 (82.0)	26 (81.2)	0.932
	≥65	11 (18.0)	6 (18.8)	
Gender	Male	39 (63.9)	23 (71.9)	0.440
	Female	22 (36.1)	9 (28.1)	
Primary tumor site	Left	32 (52.5)	20 (62.5)	0.448
	Right	28 (45.9)	11 (34.4)	
	Multisite	1 (1.6)	1 (3.1)	
Differentiation	Well or moderate	42 (68.9)	24 (75.0)	0.388
	Poor	19 (31.1)	7 (21.9)	
	Unknown	--	1 (3.1)	
Primary tumor resected	Yes	36 (59.0)	32 (100.0)	<0.001
	No	25 (41.0)	--	
Primary T stage	T3	17 (27.9)	14 (43.8)	0.103
	T4	31 (50.8)	16 (50.0)	
	Tx	13 (21.3)	2 (6.2)	
Primary lymph node status	Positive	54 (88.5)	23 (71.9)	0.043
	Negative	7 (11.5)	9 (28.1)	
Previous adjuvant chemotherapy	Yes	14 (23.0)	8 (25.0)	0.825
	No	47 (77.0)	24 (75.0)	
Metastatic disease	Synchronous	47 (77.0)	24 (75.0)	0.825
	Metachronous	14 (23.0)	8 (25.0)	
Number of organs involved	<2	19 (31.1)	27 (84.4)	<0.001
	≥2	42 (68.9)	5 (15.6)	
Oligometastatic state ^a^	OMD	1 (1.7)	10 (31.3)	<0.001
	Non-OMD	59 (98.3)	22 (68.7)	
Metastatic location(s)	Liver or lung only	6 (9.8)	14 (43.8)	<0.001
	Peritoneal only	9 (14.8)	13 (40.6)	0.005
	Distant lymph nodes only	4 (6.6)	--	0.295
	Non-isolated peritoneal metastasis ^b^	17 (27.9)	4 (12.5)	0.092
	Involved one other organ	5 (29.4)	4 (100.0)	0.021
	Involved > one other organ	12 (70.6)	--	--
	Multiple non-peritoneal metastases ^b^	25 (41.0)	1 (3.1)	<0.001
First-line chemotherapy	Tri-chemo	19 (31.2)	13 (40.6)	0.180
	Bi-chemo	39 (63.9)	15 (46.9)	
	Others	2 (3.3)	1 (3.1)	
	Unknown	1 (1.6)	3 (9.4)	
Bev in first-line chemotherapy	Yes	30 (49.2)	18 (56.2)	0.113
	No	30 (49.2)	11 (34.4)	
	Unknown	1 (1.6)	3 (9.4)	

^a^ One patient was not evaluable due to oligometastatic state. ^b^ Non-isolated peritoneal metastasis: Peritoneal metastasis and other organs metastases. Multiple non-peritoneal metastases: Combination of two or more metastatic organs, including liver, lung, distant lymph node or other non-peritoneal sites. Abbreviations: OMD, oligometastatic disease; Tri-chemo, tri-chemotherapy; Bi-chemo, bi-chemotherapy; Bev, bevacizumab; PTs, palliative treatments; LRIs, locoregional interventions.

## Data Availability

The data in favor of these findings of the current study are available from the corresponding author upon any reasonable request.

## Supplementary Materials

The following is available online at https://www.mdpi.com/article/10.3390/biom11091268/s1, Table S1: Treatment features and recurrent disease of mCRC patients with the BRAF V600E mutation who received LRIs, Table S2: Oligometastatic state, treatment features, and recurrent disease of mCRC patients with the BRAF V600E mutation in the LRIs-LLM subgroup, Figure S1: Kaplan–Meier analysis of DFS and SAR for mCRC patients with the BRAF V600E mutation who received LRIs. Abbreviations: DFS, disease-free survival; SAR, survival after recurrence.
