# Supplementary material for: The Prognostic Value of Locoregional Interventions for BRAF V600E Metastatic Colorectal Cancer: A Retrospective Cohort Analysis
Source: Biomolecules. 2021 Aug 25;11(9):1268. doi: 10.3390/biom11091268 (PMC8468777; doi:10.3390/biom11091268)
Supplement: Supplementary file 1 [file biomolecules-11-01268-s001.zip › biomolecules-1281914-supplementary.pdf]

Supplementary Materials

Table S1. Treatment features and recurrent disease of mCRC patients with the BRAF V600E mutation who received LRIs.

**Characteristics**	No. of Patients (%)
	Overall (N=32)
Metastatic location(s)	
LLM	14 (43.8)
Non-LLM	18 (56.2)
CEA level before LRIs (median, range) [a]	3.4 (0.6-186.7)
Chemotherapy before LRIs [a]	
Yes	14 (45.2)
No	17 (54.8)
Patterns of LRIs	
Surgery ± RFA/MWA	27 (84.4)
MWA [b]	3 (9.4)
Cryoablation	1 (3.1)
TACE	1 (3.1)
Chemotherapy after LRIs [a]	
Yes	25 (80.6)
No	6 (19.4)
Recurrent disease [a]	
Yes	24 (77.4)
No	7 (22.6)
Recurrent sites	
Liver or lung only	7 (29.2)
Peritoneal only	3 (12.5)
Other only	1 (4.1)
Multiple	13 (54.2)
Median number of recurrent sites (range)	2 (1.0-5.0)

[a] Presence of missing data in these variables.

[b] One patient underwent ablation of lung lesions through MWA and stereotactic body radiation therapy.

Abbreviations: LLM, liver-limited or lung-limited metastasis; CEA, carcinoembryonic antigen; LRIs, locoregional interventions; RFA, radiofrequency ablation; MWA, microwave ablation; TACE, transcatheter arterial chemoembolization.

Table S2. Oligometastatic state, treatment features, and recurrent disease of mCRC patients with the BRAF V600E mutation in the LRIs-LLM subgroup.

Patients	Oligometastatic state	OMD/Low TB	Chemotherapy before LRIs	Objective response	Chemotherapy after LRIs	DFS (months)	First site(s) of recurrence
1	OMD	Yes	Bev+FOLFOXIRI	PR	Bev+ Capecitabine	2.2	**NED**
2	OMD	NE	No	--	Bev+FOLFOX	2.7	Liver
3	Non-OMD	--	XELOX+radiotherapy	SD	XELOX	1.1	Liver

4	Non-OMD	--	Bev+FOLFOXIRI	PR	FOLFOXIRI	5.0	Liver, anastomotic recurrence
5	OMD	No	FOLFOX	PR	FOLFOX+HAI (FUDR)	16.6	Lung
6	OMD	No	FOLFIRI	SD	FOLFIRI	13.6	Liver, lung, distant lymph nodes
7	OMD	Yes	No	--	--	27.0	Para-aortic lymph nodes
8	OMD	Yes	XELOX	SD	--	**26.6**	**NED**
9	Non-OMD	--	Bev+FOLFOXIRI	PR	Bev+FOLFOXIRI	4.4	**NED**
10	Non-OMD	--	Bev+FOLFOXIRI	PR	Capecitabine	3.3	Liver
11	OMD	Yes	Bev+FOLFOXIRI	PR	Bev+ Capecitabine	**21.0**	**NED**
12	OMD	Yes	No	--	--	8.4	Lung
13	OMD	Yes	Bev+FOLFOXIRI	SD	--	7.2	**NED**
14	OMD	Yes	No	--	Tegafur	12.4	Brain, left adrenal gland, distant lymph nodes

Abbreviations: OMD, oligometastatic disease; TB, tumor burden; NE, not evaluated; Bev, bevacizumab; PR, partial response; SD, stable disease; HAI, hepatic arterial infusion; NED, no evidence of disease.

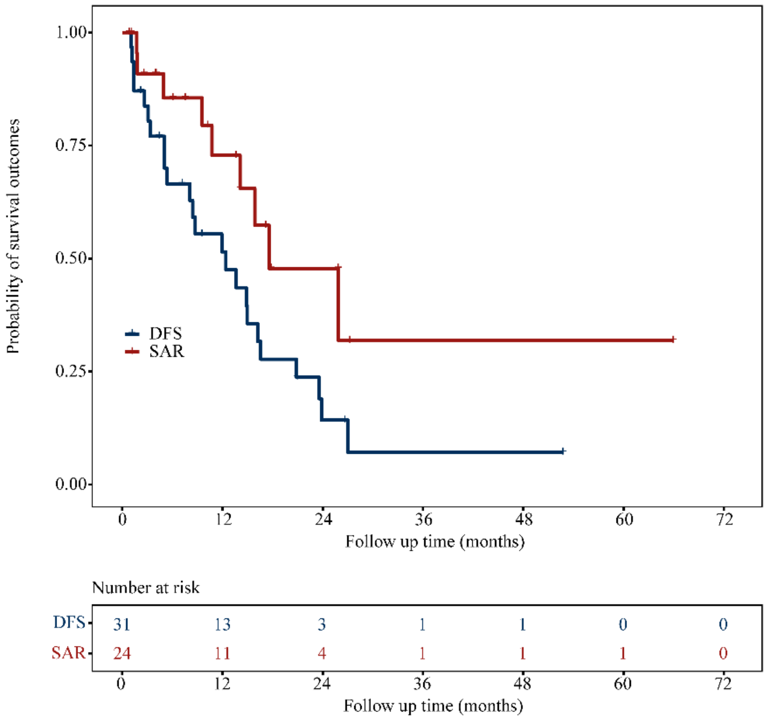

Figure S1. Kaplan-Meier analysis of DFS and SAR for mCRC patients with the BRAF V600E mutation who received LRIs. Abbreviations: DFS, disease-free survival; SAR, survival after recurrence.
